# Author Correction: Neuroinvasion and anosmia are independent phenomena upon infection with SARS-CoV-2 and its variants

**DOI:** 10.1038/s41467-023-42800-7

**Published:** 2023-12-04

**Authors:** Guilherme Dias de Melo, Victoire Perraud, Flavio Alvarez, Alba Vieites-Prado, Seonhee Kim, Lauriane Kergoat, Anthony Coleon, Bettina Salome Trüeb, Magali Tichit, Aurèle Piazza, Agnès Thierry, David Hardy, Nicolas Wolff, Sandie Munier, Romain Koszul, Etienne Simon-Lorière, Volker Thiel, Marc Lecuit, Pierre-Marie Lledo, Nicolas Renier, Florence Larrous, Hervé Bourhy

**Affiliations:** 1 Institut Pasteur, Université Paris Cité, Lyssavirus Epidemiology and Neuropathology Unit, F-75015 Paris, France; 2 Institut Pasteur, Université Paris Cité, Channel Receptors Unit, F-75015 Paris, France; 3 Sorbonne Université, Collège Doctoral, F-75005 Paris, France; 4 Institut du Cerveau et de la Moelle Épinière, Laboratoire de Plasticité Structurale, Sorbonne Université, INSERM U1127, CNRS UMR7225, 75013 Paris, France; 5 Institute of Virology and Immunology (IVI), Bern, Switzerland; Department of Infectious Diseases and Pathobiology, Vetsuisse Faculty, University of Bern, Bern, Switzerland; 6 Institut Pasteur, Université Paris Cité, Histopathology Platform, F-75015 Paris, France; 7 Institut Pasteur, Université Paris Cité, Spatial Regulation of Genomes Laboratory, F-75015 Paris, France; 8 Institut Pasteur, Université Paris Cité, Molecular Genetics of RNA viruses Unit, F-75015 Paris, France; 9 Institut Pasteur, Université Paris Cité, Evolutionary Genomics of RNA Viruses Group, F-75015 Paris, France; 10 Multidisciplinary Center for Infectious Diseases, University of Bern, Bern, Switzerland; 11 Institut Pasteur, Université Paris Cité, Inserm U1117, Biology of Infection Unit, 75015 Paris, France; 12 Necker-Enfants Malades University Hospital, Division of Infectious Diseases and Tropical Medicine, APHP, Institut Imagine 75006 Paris, France; 13 Institut Pasteur, Université Paris Cité, Perception and Memory Unit, F-75015 Paris, France; CNRS UMR3571, 75015 Paris, France

**Keywords:** SARS-CoV-2, Olfactory bulb, Infection

Correction to: *Nature Communications* 10.1038/s41467-023-40228-7, published online 26 July 2023

The original version of this Article contained an error in the summary of the presented work in the discussion section.

The original version read “Here we show that all evaluated SARS-CoV-2 viruses (Wuhan, Gamma, Delta and Omicron/BA.1), including novel generated recombinant SARS-CoV-2, are able to invade the brain, most likely via the olfactory nerves, and of promoting inflammation in the nervous tissue.”

This has been amended to “Here we show that all evaluated SARS-CoV-2 viruses (Wuhan, Gamma, Delta and Omicron/BA.1), including novel generated recombinant SARS-CoV-2, are able to cause neuroinflammation following brain invasion. This is most likely via the olfactory bulb; however, the exact nerval route warrants further research.”

This has now been corrected in both the PDF and HTML versions of the Article.

