# Supplementary material for: Neuroinvasion and anosmia are independent phenomena upon infection with SARS-CoV-2 and its variants
Source: Nat Commun. 2023 Jul 26;14:4485. doi: 10.1038/s41467-023-40228-7 (PMC10372078; doi:10.1038/s41467-023-40228-7)
Supplement: Supplementary file 1 — Supplementary information [file 41467_2023_40228_MOESM1_ESM.pdf]

Neuroinvasion and anosmia are independent phenomena upon infection with SARS-CoV-2 and its variants

SUPPLEMENTARY INFORMATION

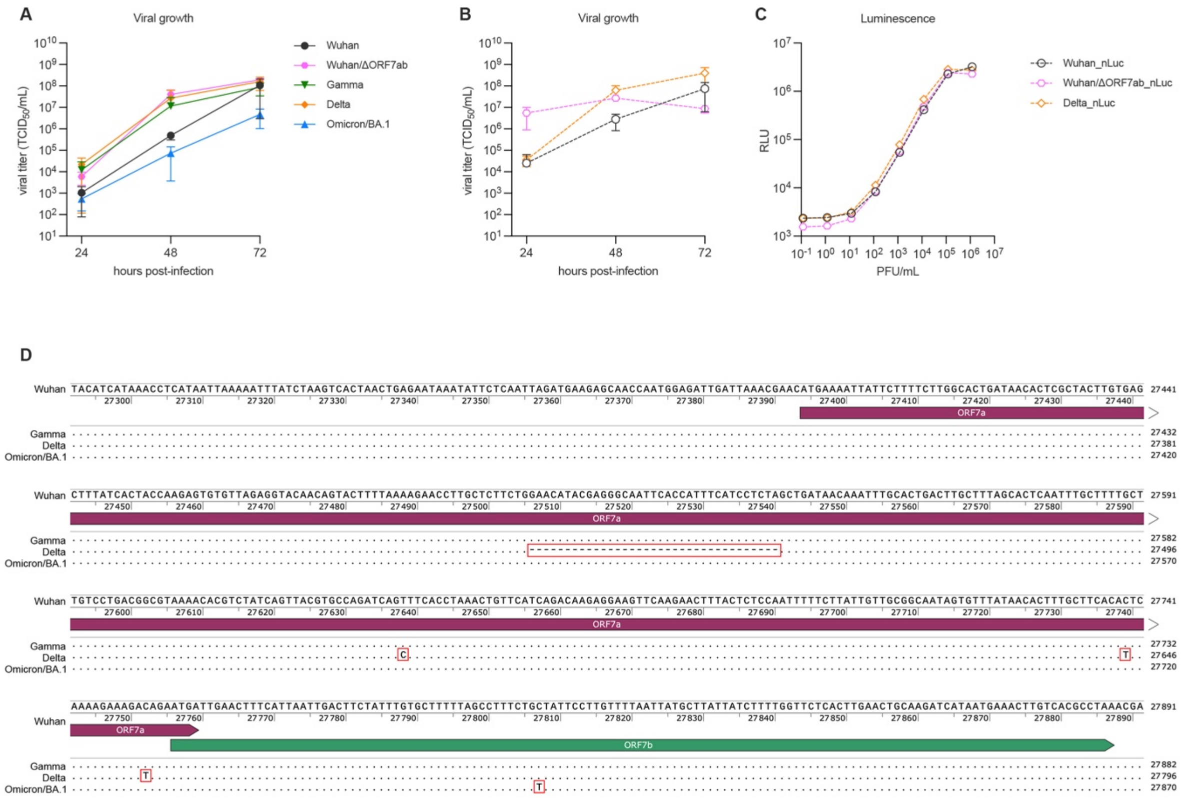

Supplementary Fig. 1. ***In vitro*** **viral growth curves of different SARS-CoV-2 viruses. A.** Growth curves of the SARS-CoV-2 original virus (Wuhan), the recombinant Wuhan/ΔORF7ab, or the variants of concern (VoC) Gamma, Delta and Omicron/BA.1. **B.** Growth curves of the recombinant nanoluciferase-expressing SARS-CoV-2 viruses Wuhan_nLuc, Wuhan/ΔORF7ab_nLuc, and Delta_nLuc. A-B: Horizontal lines indicate median and the interquartile range (n=3 independent replicates/time-point). Viral titers are expressed as the median tissue culture infectious dose ($TCID_{50}$)/mL. **C**. Dilution curves showing the luminescence activity of the recombinant nanoluciferase-expressing SARS-CoV-2 viruses Wuhan_nLuc, Wuhan/ΔORF7ab_nLuc, and Delta_nLuc. RLU: relative light units. **D**. Multiple sequence alignment of the ORF7ab regions of Gamma, Delta and Omicron/BA.1 in comparison with the SARS-CoV-2 original virus (Wuhan). Deletions and mutations are indicated inside red squares. Related to Fig. 1, 3, and 4.

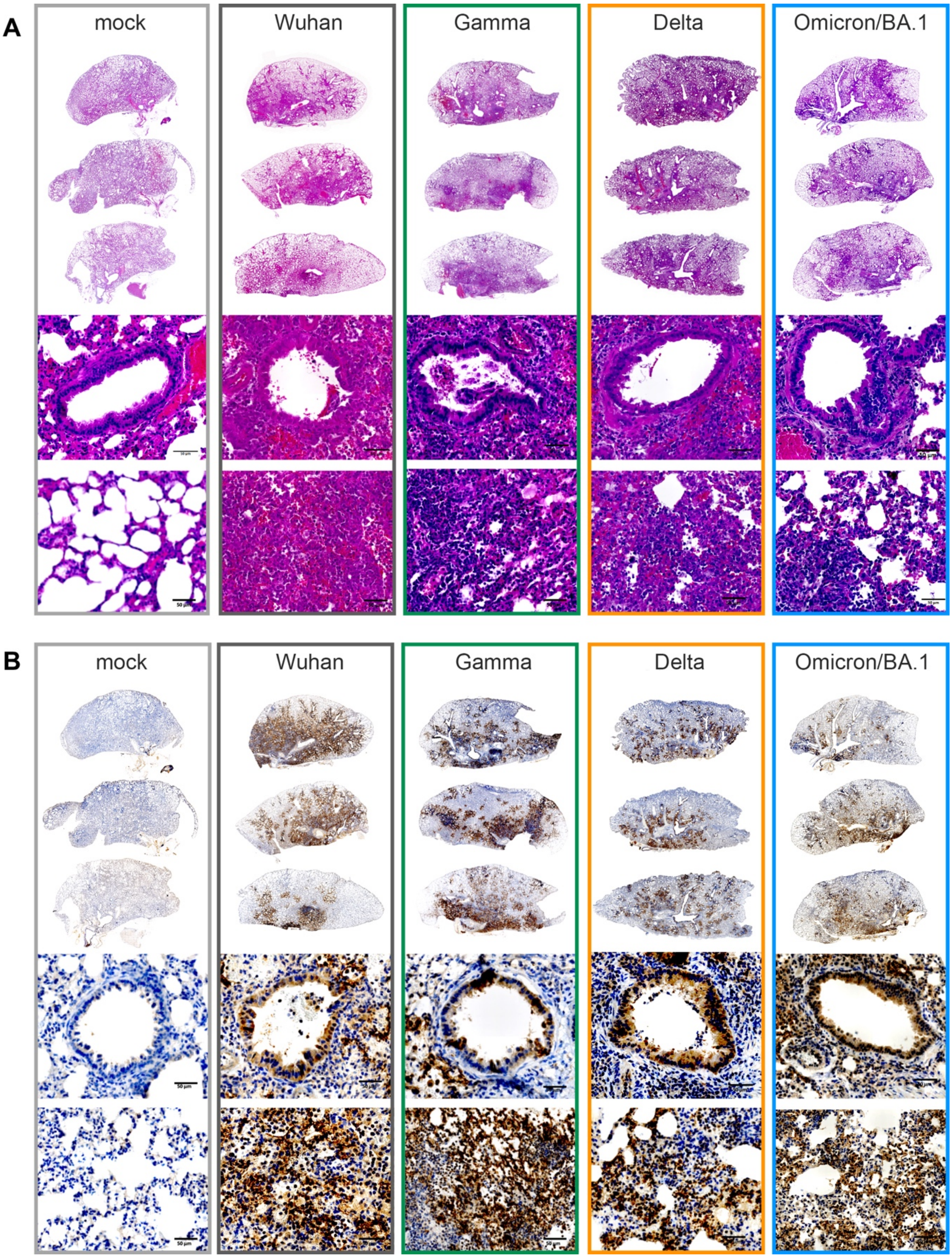

Supplementary Fig. 2. Histopathology and immunohistochemical aspects of freshly-collected lungs from hamsters infected with SARS-CoV-2 original virus (Wuhan) or the variants of concern (VoC) Gamma, Delta and Omicron/BA.1 at 4 days post-infection (dpi). A. Representative images of Hematoxylin and Eosin (H&E) stained-whole lung sections (upper panels), bronchiolar epithelium (middle panels) and alveoli (bottom panels). **B.** Representative images of whole lung sections (upper panels), bronchiolar epithelium (middle panels) and alveoli (bottom panels) immuno-stained with SARS-CoV-2 N antibody. Immunoperoxidase, scale bar = 50 µm (n=8/group, except Omicron/BA.1 where n=4). Related to Fig. 1.

Neuroinvasion and anosmia are independent phenomena upon infection with SARS-CoV-2 and its variants

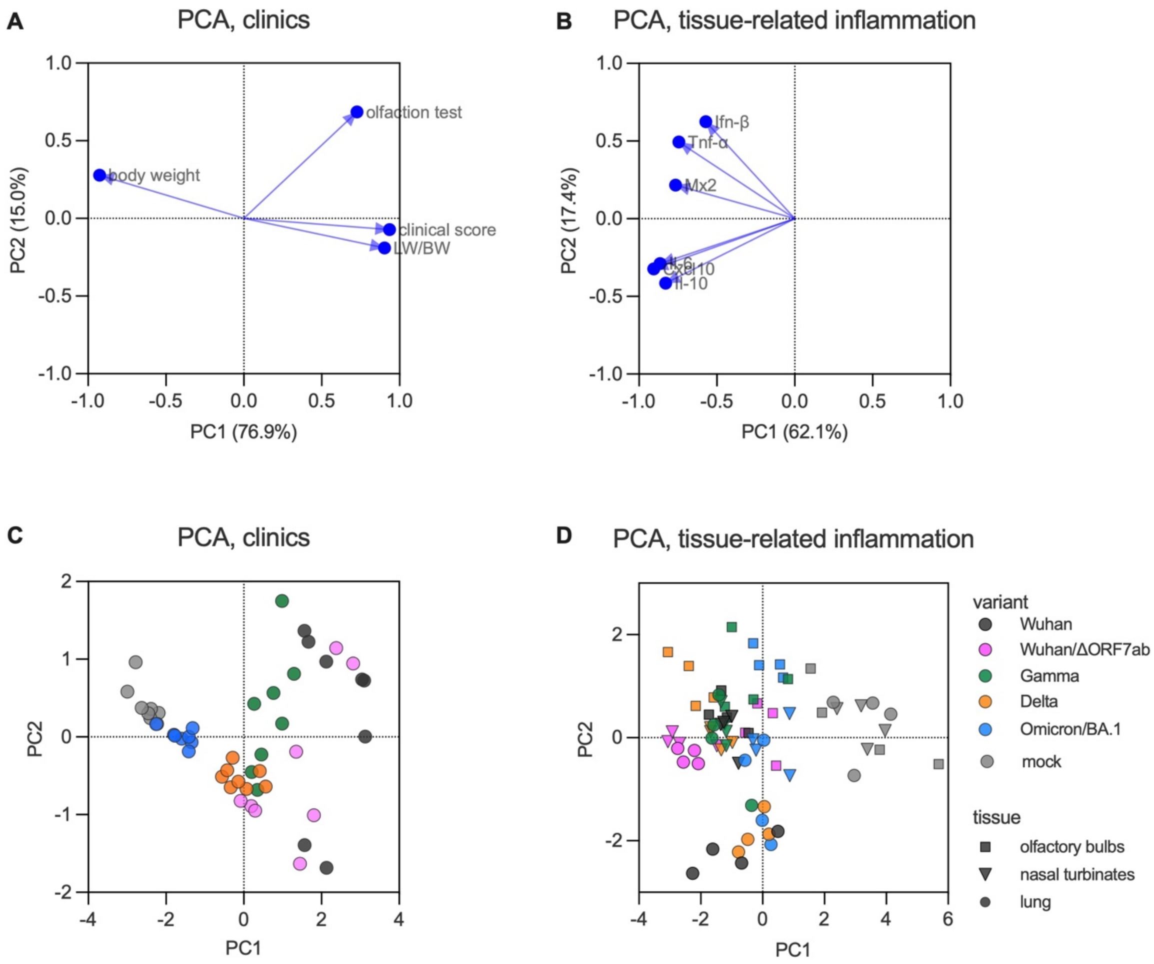

Supplementary Fig. 3. Principal component analysis (PCA) of clinical parameters and immune mediators in different tissues of hamsters infected with SARS-CoV-2 original virus (Wuhan), the recombinant Wuhan/ΔORF7ab or the variants of concern (VoC) Gamma, Delta and Omicron/BA.1. A. Variable correlation plot showing the correlation of clinical parameters in hamsters at 4 days post-infection (body weight variation, clinical score, olfaction test and lung weight-to-body weight ratio 'LW/BW'). The two-first principal components explained 92.2% of sample variability (n=8/group). **B.** Variable correlation plot showing the correlation of immune mediators' gene expression (*Mx2, Ifn*-β, *Il-6, Cxcl10, Tnf*-α and *Il-10*) in the olfactory bulb, nasal turbinates and lungs of hamsters at 4 days post-infection. The two-first principal components explained 80.5% of sample variability (n=4/group). **CD.** PCA plots. Each symbol represents one animal, colored according to the virus variant. Related to Fig. 1 and 2.

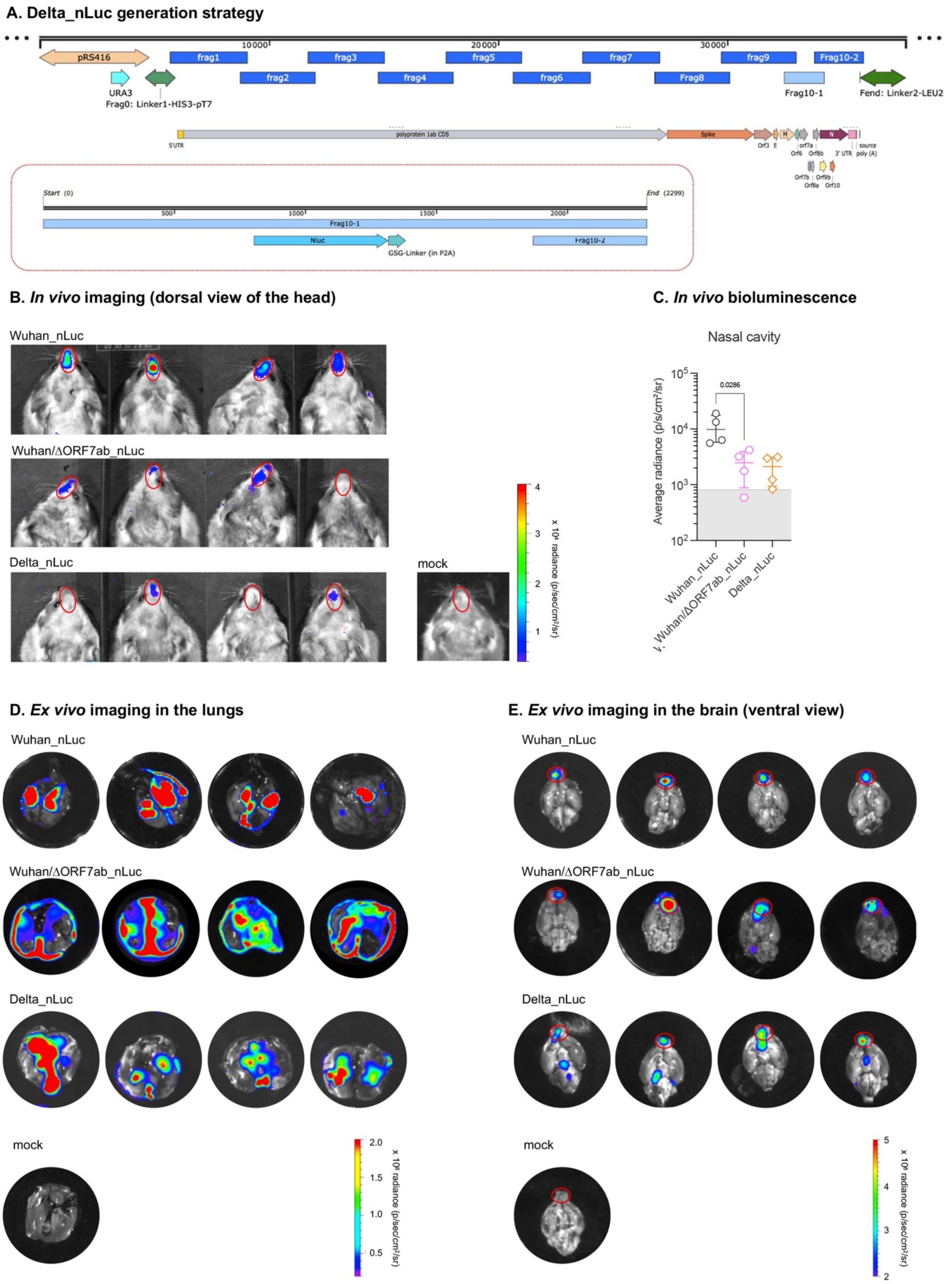

Supplementary Fig. 4. *In vivo* and *ex vivo* imaging in hamsters infected with bioluminescent recombinant SARS-CoV-2 viruses at 4 days post-infection (dpi). **A.** Delta_nLuc generation strategy. **B.** *In vivo* imaging of hamsters infected with the recombinant viruses Wuhan_nLuc and Wuhan/ΔORF7ab_nLuc (based on the original SARS-CoV-2 Wuhan) and Delta_nLuc (based on the Delta). The regions of interest (ROIs) are shown as red circles (n=4/group). **C.** *In vivo* bioluminescence values from the nasal cavity (n=4/group). Horizontal lines indicate median and the interquartile range.

Neuroinvasion and anosmia are independent phenomena upon infection with SARS-CoV-2 and its variants

Kruskal-Wallis test followed by the Dunn's multiple comparisons test (the adjusted p value is indicated when significant). The gray crosshatched area corresponds to background signals obtained from a mock-infected hamster. **DE.** *Ex vivo* imaging of lungs (D) and brains (E) from hamsters infected with Wuhan_nLuc, Wuhan/ΔORF7ab_nLuc and Delta_nLuc and mock-infected at 4 dpi. The regions of interest (ROIs) are shown as red circles in the olfactory bulbs (for the lungs and the whole brain, the values from the whole organ were acquired). Related to Fig. 3.

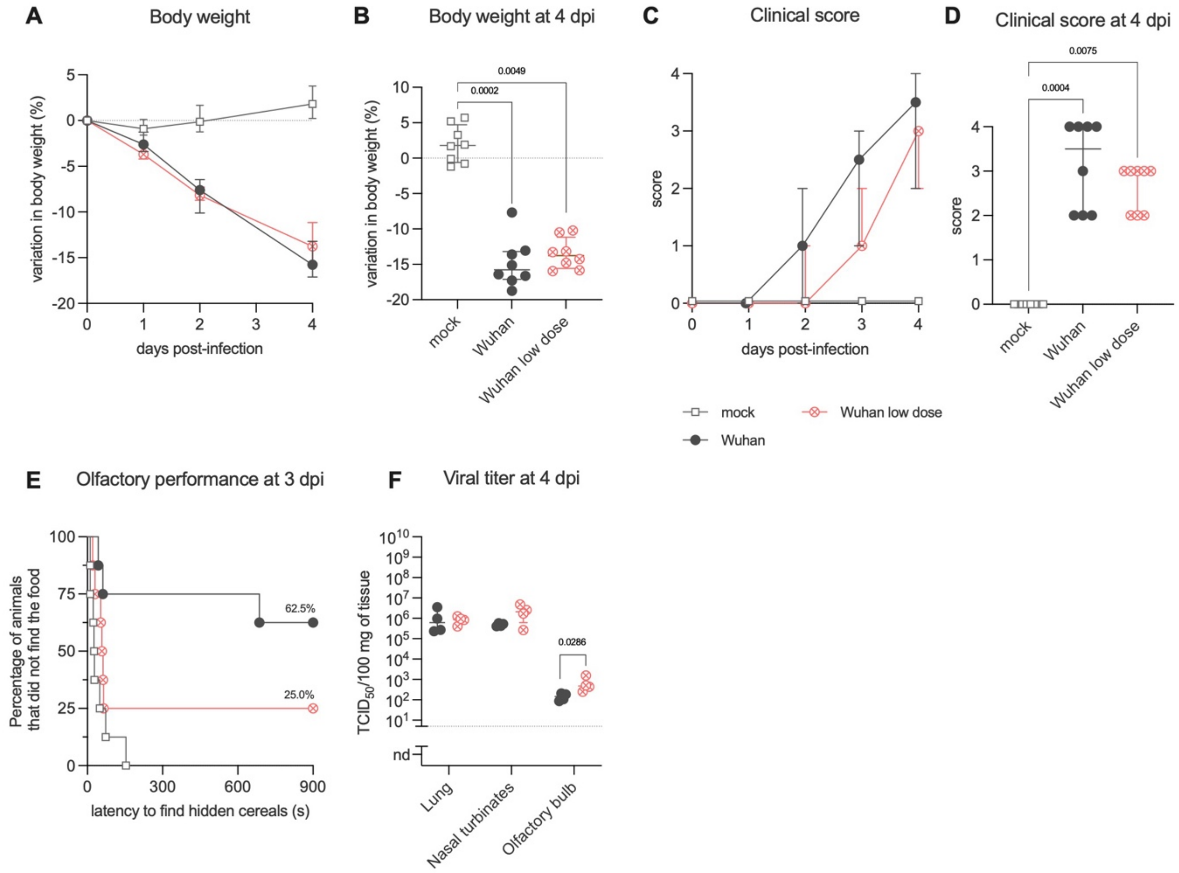

Supplementary Fig. 5. Clinical profile of hamsters inoculated with SARS-CoV-2 original virus (Wuhan) with a regular infectious dose ($6x10^4$ PFU) and with a low infectious dose ($6x10^2$ PFU). AB. Body weight variation over 4 days post-infection (dpi). B: Kruskal-Wallis test followed by the Dunn's multiple comparisons test (the adjusted p value is indicated when significant). **CD.** Clinical score comparison in the acute phase (4 dpi). The clinical score is based on a cumulative 0–4 scale: ruffled fur; slow movements; apathy; and absence of exploration activity. D: Kruskal-Wallis test followed by the Dunn's multiple comparisons test (the adjusted p value is indicated when significant). A-D: Horizontal lines indicate median and the interquartile range (n=8/group). **E**. Olfactory performance measured at 3 dpi. The olfaction test is based on the hidden (buried) food finding test. Curves represent the olfactory performance of animals during the test (n=8/group). **F**. Infectious viral titers in the lung, nasal turbinates, and olfactory bulbs at 4 dpi expressed as $TCID_{50}$ per 100 mg of tissue. Horizontal lines indicate median and the interquartile range (n=4/group). Mann-Whitney test (the p value is indicated when significant; two-sided). Data for SARS-CoV-2 Wuhan already presented in Fig. 1 and 2.

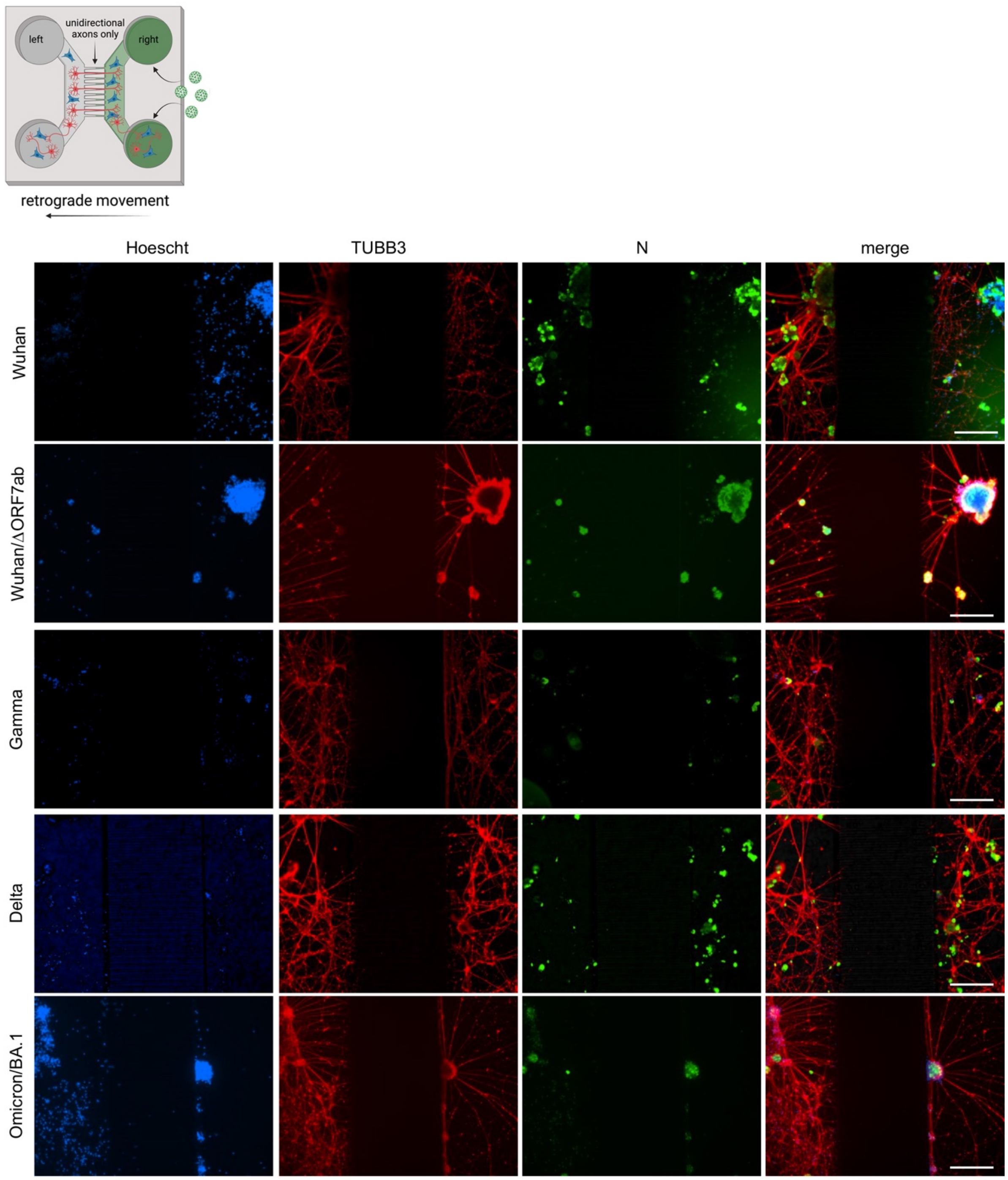

Supplementary Fig. 6. SARS-CoV-2 travels retrogradely along axons in *in vitro* neuron-epithelial networks. Schematic view of an axon diode in a microfluidic device where the left and the right chambers are connected exclusively by axons. In these images, SARS-CoV-2 was added in the right chamber**:** Wuhan, Wuhan/ΔORF7ab, Gamma, Delta, and Omicron/BA.1, showing infected cells in both the left and right chambers (scale bar = 200 µm). Hoechst: nuclei (blue). TUBB3: β-Tubulin III (red). N: SARS-CoV-2 nucleocapsid (green). The black zones in the central part of the photomicrographs corresponds to the microchannels, which are not accessible during immunostainings. These photomicrographs are representative of 3 independent experiments. Related to Fig. 4. The top left panel was created with BioRender.com.

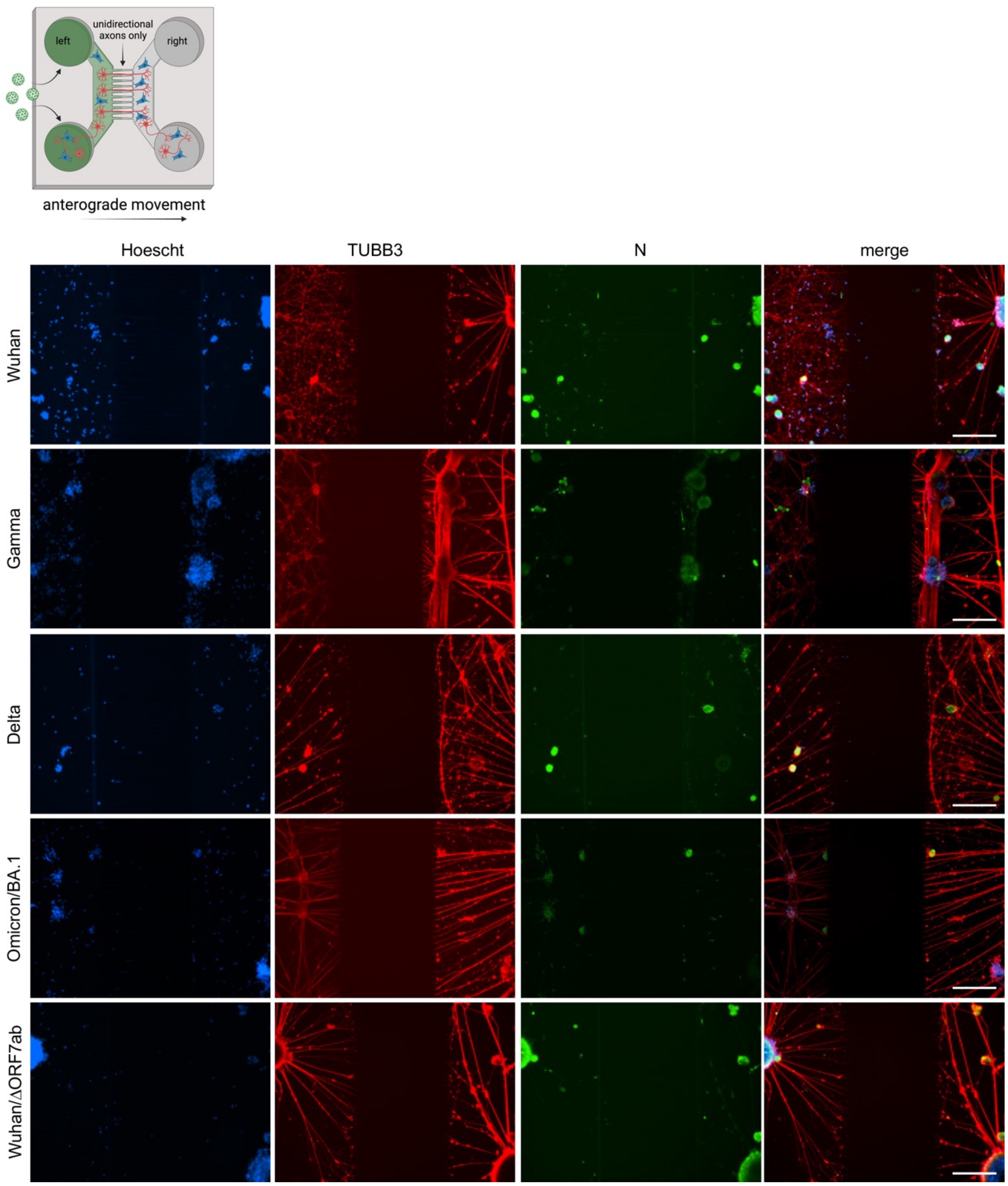

Supplementary Fig. 7. SARS-CoV-2 travels anterogradely along axons in *in vitro* neuron-epithelial networks. Schematic view of an axon diode in a microfluidic device where the left and the right chambers are connected exclusively by axons. In these images, SARS-CoV-2 was added in the left chamber**:** Wuhan, Wuhan/ΔORF7ab, Gamma, Delta, and Omicron/BA.1, showing infected cells in both the left and right chambers (scale bar = 200 µm). Hoechst: nuclei (blue). TUBB3: β-Tubulin III (red). N: SARS-CoV-2 nucleocapsid (green). The black zones in the central part of the photomicrographs corresponds to the microchannels, which are not accessible during immunostainings. These photomicrographs are representative of 3 independent experiments. Related to Fig. 4. The top left panel was created with BioRender.com.

Neuroinvasion and anosmia are independent phenomena upon infection with SARS-CoV-2 and its variants

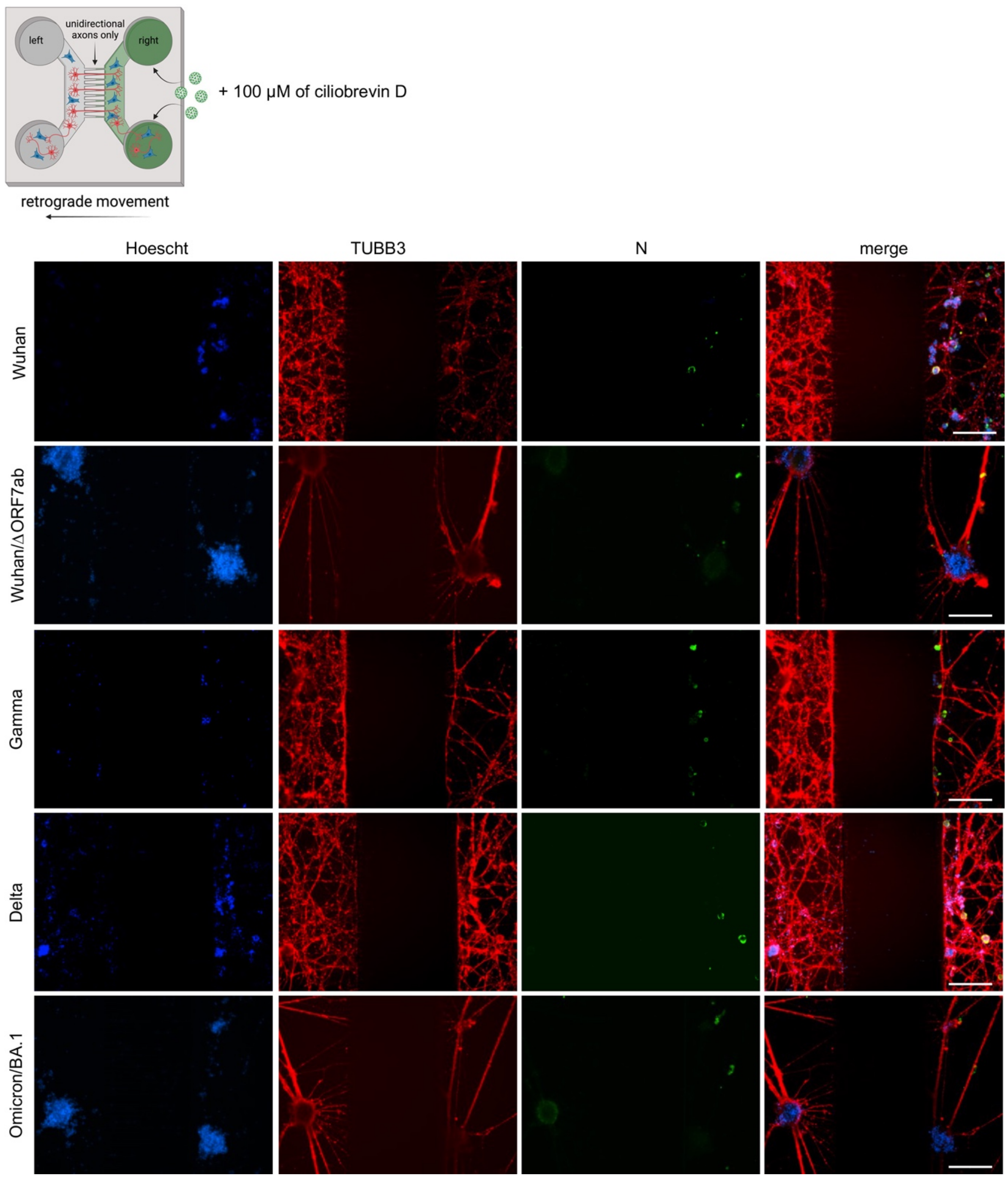

Supplementary Fig. 8. SARS-CoV-2 retrograde movement inside axons can be blocked by ciliobrevin D in *in vitro* neuron-epithelial networks. Schematic view of an axon diode in a microfluidic device where the left and the right chambers are connected exclusively by axons. In these images, 100 µM of ciliobrevin was added in both chambers and SARS-CoV-2 was added in the right chamber**:** Wuhan, Wuhan/ΔORF7ab, Gamma, Delta, and Omicron/BA.1, showing infected cells only in the right chambers (scale bar = 200 µm). Hoechst: nuclei (blue). TUBB3: β-Tubulin III (red). N: SARS-CoV-2 nucleocapsid (green). The black zones in the central part of the photomicrographs corresponds to the microchannels, which are not accessible during immunostainings. These photomicrographs are representative of 3 independent experiments. Related to Fig. 4. The top left panel was created with BioRender.com.

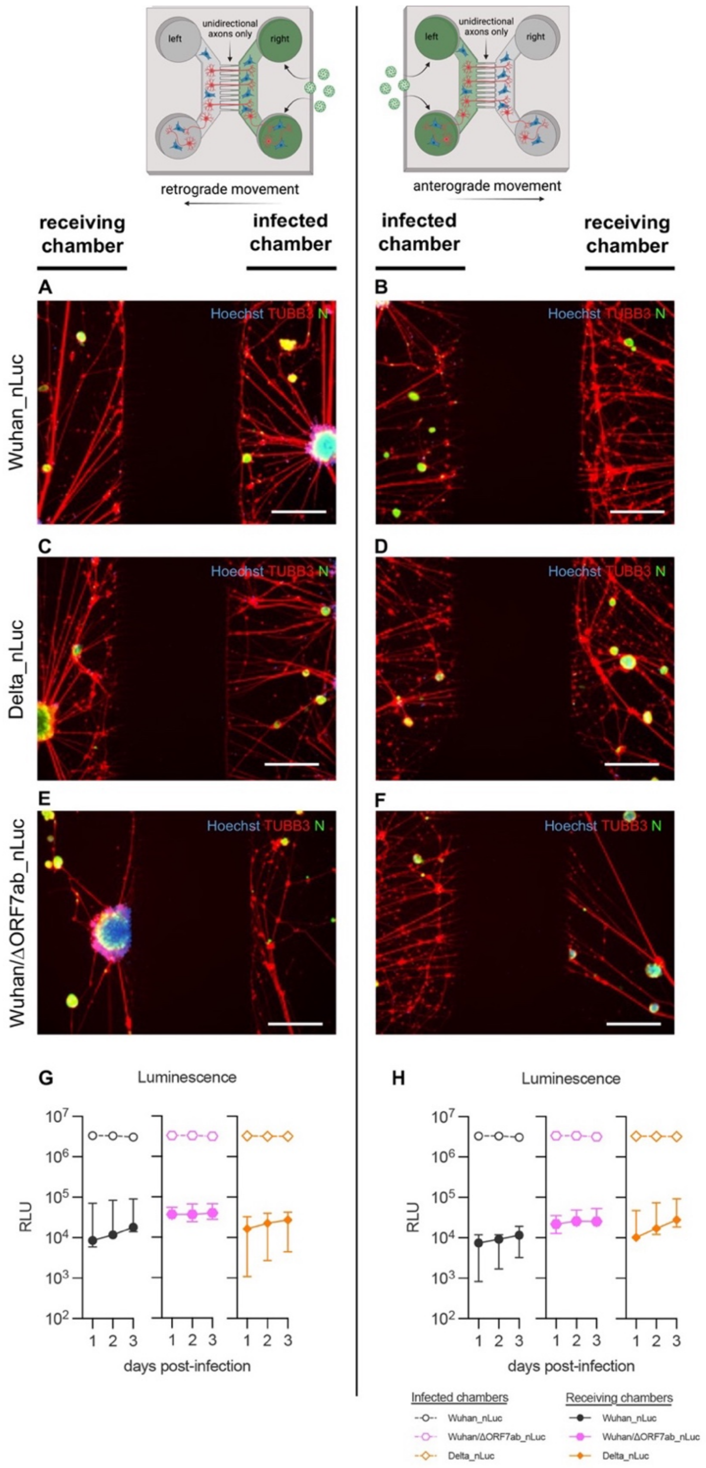

Supplementary Fig. 9. Quantification of the intra-axonal traffic of SARS-CoV-2 in *in vitro* neuron-epithelial networks. AF. Photomicrographs of retrograde (A,C,E) and anterograde (B, D, F) movement of the recombinant viruses Wuhan_nLuc (AB), Wuhan/∆ORF7ab_nLuc (CD) and Delta_nLuc (EF) at 3 dpi (scale bar = 200 µm). Hoechst: nuclei (blue). TUBB3: β-Tubulin III (red). N: SARS-CoV-2 nucleocapsid (green). A-F: These photomicrographs are representative of 3 independent experiments. **GH.** SARS-CoV-2 quantification by sequential luminescence measurements in the supernatants, in the retrograde (G) or anterograde (H) directions. Horizontal lines indicate median and the interquartile range (n=3 independent replicates). Open symbols indicate the infected chambers, full-colored symbols indicate the chambers receiving the virus via the axons. RLU: relative light units. Related to Fig. 4. The top panels were created with BioRender.com.

Supplementary Table 1. Primer sequences used to amplify the different fragments for yeast recombination.

Fragment	Sequence (5'-3') *		Position on the viral genome	Size
pRS416	Forward	CCAGTAATGTGGACATTGCC	/	4816
	Reverse	CATCAACGACCTTGTCTTCAGTA	/	
Linker His3	Forward	CCTGAAAGCACTTGAAGAATTCC	/	1448
	Reverse	GGTATGCTAAGGCACAGCACACT	78	
Frag1	Forward	GCCATTCATCCACAGTTGACA	in *HIS*3	3213
	Reverse	CATGGTGCTGACAGTGGAGTCT	3183	
Frag2	Forward	TGAGCCATCGTGCCAATG	2885	3251
	Reverse	AGCCCGTCTGCTGGTATCAC	6135	
Frag3	Forward	GGTTGCCAAACCTTATCAGAAATG	5804	3339
	Reverse	TTCACCTGTTCCACAGCCTTG	9142	
Frag4	Forward	ACAGAGAGAAGATGACGCAGATAATG	8885	3251
	Reverse	GCCTGAATGGCCACGTACA	12135	
Frag5	Forward	TGCGGATGATATCTCAACTTTAACTG	11824	3311
	Reverse	AAAGGAAAGCAAAGTTTGTATTGTCA	15134	
Frag6	Forward	TGCGGATGATATCTCAACTTTAACTG	14782	3350
	Reverse	AAAGGAAAGCAAAGTTTGTATTGTCA	18131	
Frag7	Forward	TGCGGATGATATCTCAACTTTAACTG	17786	3371
	Reverse	AAAGGAAAGCAAAGTTTGTATTGTCA	21156	
Frag8	Forward	TGCGGATGATATCTCAACTTTAACTG	20932	3276
	Reverse	AAAGGAAAGCAAAGTTTGTATTGTCA	24207	
Frag9	Forward	TGCGGATGATATCTCAACTTTAACTG	23854	3253
	Reverse	AAAGGAAAGCAAAGTTTGTATTGTCA	28832	
Frag10-1	Forward	TGCGGATGATATCTCAACTTTAACTG	26589	1720
nLuc	Reverse	AAAGGAAAGCAAAGTTTGTATTGTCA	28332	
Frag10-2	Forward	TGCGGATGATATCTCAACTTTAACTG	27903	2132
	Reverse	AAAGGAAAGCAAAGTTTGTATTGTCA	in *LEU*2	
Linker Leu2	Forward	TGCGGATGATATCTCAACTTTAACTG	/	2041
	Reverse	AAAGGAAAGCAAAGTTTGTATTGTCA	/	

*Based on the sequence of SARS-CoV-2 Delta/2021/I7.2 200 (GISAID ID: EPI_ISL_2029113) [1]

Neuroinvasion and anosmia are independent phenomena upon infection with SARS-CoV-2 and its variants

Supplementary Table 2. Primer sequences used for qPCR in the golden hamster tissues.

Gene	Sequence (5'-3')		Reference
	Forward	CCAGTAATGTGGACATTGCC	
Mx2	Reverse	CATCAACGACCTTGTCTTCAGTA	[2]
	Probe	FAM-TGTCCACCAGATCAGGCTTGGTCA-TAMRA	
	Forward	ACCCTAAAGGAAGTGCCAGA	
Ifn-β	Reverse	CCAGCTGCCAGTAATAGCTC	[3]
	Probe	FAM-AGTTTGACTACAAGGATTAGCTTGAA-TAMRA	
	Forward	CCTGAAAGCACTTGAAGAATTCC	
Il-6	Reverse	GGTATGCTAAGGCACAGCACACT	[2]
	Probe	FAM-AGAAGTCACCATGAGGTCTACTCGGCAAAA-TAMRA	
	Forward	GCCATTCATCCACAGTTGACA	
Cxcl10	Reverse	CATGGTGCTGACAGTGGAGTCT	[2]
	Probe	FAM-CGTCCCGAGCCAGCCAACGA-TAMRA	
	Forward	TGAGCCATCGTGCCAATG	
Tnf-α	Reverse	AGCCCGTCTGCTGGTATCAC	[4]
	Probe	FAM-CGGCATGTCTCTCAAAGACAACCAG-TAMRA	
	Forward	GGTTGCCAAACCTTATCAGAAATG	
Il-10	Reverse	TTCACCTGTTCCACAGCCTTG	[4]
	Probe	FAM-TGCAGCGCTGTCATCGATTTCTCCC-TAMRA	
	Forward	ACAGAGAGAAGATGACGCAGATAATG	
γ-actin	Reverse	GCCTGAATGGCCACGTACA	[4]
	Probe	FAM-TTG AAA CCT TCA ACA CCC CAG CC-TAMRA	
	Forward	TGCGGATGATATCTCAACTTTAACTG	
Hprt	Reverse	AAAGGAAAGCAAAGTTTGTATTGTCA	[2]
	Probe	FAM-AAAGAATGTCTTGATTGTTGAAGGTAAAACTGACATTGG-TAMRA	

REFERENCES

1. Planas, D., *et al.* Reduced sensitivity of SARS-CoV-2 variant Delta to antibody neutralization. *Nature* **596**, 276-280 (2021).
2. Zivcec, M., Safronetz, D., Haddock, E., Feldmann, H. & Ebihara, H. Validation of assays to monitor immune responses in the Syrian golden hamster (Mesocricetus auratus). *J Immunol Methods* **368**, 24-35 (2011).
3. Gowen, B.B., *et al.* MP-12 virus containing the clone 13 deletion in the NSs gene prevents lethal disease when administered after Rift Valley fever virus infection in hamsters. *Front Microbiol* **6**(2015).
4. Espitia, C.M., *et al.* Duplex real-time reverse transcriptase PCR to determine cytokine mRNA expression in a hamster model of New World cutaneous leishmaniasis. *BMC Immunology* **11**, 31 (2010).
