# Supplementary material for: Neuroinvasion and anosmia are independent phenomena upon infection with SARS-CoV-2 and its variants
Source: Nat Commun. 2023 Jul 26;14:4485. doi: 10.1038/s41467-023-40228-7 (PMC10372078; doi:10.1038/s41467-023-40228-7)
Supplement: Supplementary file 3 — Description of Additional Supplementary Files [file 41467_2023_40228_MOESM3_ESM.pdf]

Description of Additional Supplementary Files

Supplementary Movie 1. Light sheet imaging in the nasal turbinates and in the olfactory bulbs. 3D distribution of the nucleocapsid in the nostrils, including the olfactory mucosa, in the olfactory nerve and in neurons in the olfactory bulbs of a SARS-CoV-2 Wuhan infected hamster using whole-head clearing. Related to Fig. 3.
