# Supplementary material for: Neuroinvasion and anosmia are independent phenomena upon infection with SARS-CoV-2 and its variants
Source: Nat Commun. 2023 Jul 26;14:4485. doi: 10.1038/s41467-023-40228-7 (PMC10372078; doi:10.1038/s41467-023-40228-7)
Supplement: Supplementary file 5 — Reporting Summary [file 41467_2023_40228_MOESM5_ESM.pdf]

nature portfolio

Corresponding author(s): Hervé Bourhy

Last updated by author(s): Jun 28, 2023

Reporting Summary

Nature Portfolio wishes to improve the reproducibility of the work that we publish. This form provides structure for consistency and transparency in reporting. For further information on Nature Portfolio policies, see our Editorial Policies and the Editorial Policy Checklist.

Statistics

For all statistical analyses, confirm that the following items are present in the figure legend, table legend, main text, or Methods section.

n/a	Confirmed	
☐	☒	The exact sample size (*n*) for each experimental group/condition, given as a discrete number and unit of measurement
☐	☒	A statement on whether measurements were taken from distinct samples or whether the same sample was measured repeatedly
☐	☒	The statistical test(s) used AND whether they are one- or two-sided<br>*Only common tests should be described solely by name; describe more complex techniques in the Methods section.*
☒	☐	A description of all covariates tested
☐	☒	A description of any assumptions or corrections, such as tests of normality and adjustment for multiple comparisons
☐	☒	A full description of the statistical parameters including central tendency (e.g. means) or other basic estimates (e.g. regression coefficient) AND variation (e.g. standard deviation) or associated estimates of uncertainty (e.g. confidence intervals)
☐	☒	For null hypothesis testing, the test statistic (e.g. *F*, *t*, *r*) with confidence intervals, effect sizes, degrees of freedom and *P* value noted<br>*Give P values as exact values whenever suitable.*
☒	☐	For Bayesian analysis, information on the choice of priors and Markov chain Monte Carlo settings
☒	☐	For hierarchical and complex designs, identification of the appropriate level for tests and full reporting of outcomes
☒	☐	Estimates of effect sizes (e.g. Cohen's *d*, Pearson's *r*), indicating how they were calculated

Our web collection on statistics for biologists contains articles on many of the points above.

Software and code

Policy information about availability of computer code

Data collection	qPCR: QuantStudio Real-time PCR System (v.1.2), Applied Biosystems<br>Immunofluorescence: EVOS FL cell imaging system, Thermo Scientific<br>LSIM: ImSpector software (v.7.0.127.0), Lavision Biotec GmbH<br>Bioluminescence (in vivo): Living Image (v.4.7.4), PerkinElmer<br>Histology: Zen software (v.2.6), Zeiss<br>Luminescence (in vitro): Victor Nivo control software (v.4.0.7), PerkinElmer
Data analysis	LSIM: Bitplane Imaris (v.9.2), Oxford instruments<br>Statistical analysis: Prism 9 (v.9.5.1), GraphPad

For manuscripts utilizing custom algorithms or software that are central to the research but not yet described in published literature, software must be made available to editors and reviewers. We strongly encourage code deposition in a community repository (e.g. GitHub). See the Nature Portfolio guidelines for submitting code & software for further information.

Data

Policy information about availability of data

All manuscripts must include a data availability statement. This statement should provide the following information, where applicable:

- Accession codes, unique identifiers, or web links for publicly available datasets
- A description of any restrictions on data availability
- For clinical datasets or third party data, please ensure that the statement adheres to our policy

All relevant data supporting the key findings of this study are available within the main manuscript and its Supplementary Information. The processed data generated in this study are provided in the Source Data file.

Human research participants

Policy information about studies involving human research participants and Sex and Gender in Research.

Reporting on sex and gender	n/a
Population characteristics	n/a
Recruitment	n/a
Ethics oversight	n/a

Note that full information on the approval of the study protocol must also be provided in the manuscript.

Field-specific reporting

Please select the one below that is the best fit for your research. If you are not sure, read the appropriate sections before making your selection.

☒ Life sciences ☐ Behavioural & social sciences ☐ Ecological, evolutionary & environmental sciences

For a reference copy of the document with all sections, see nature.com/documents/nr-reporting-summary-flat.pdf

Life sciences study design

All studies must disclose on these points even when the disclosure is negative.

Sample size	We use the minimum number of animals necessary to achieve statistical significance (n=8 for behavioral tests / n=4 for molecular tests) based on previous data observed using this model.<br><br>1. de Melo GD, Lazarini F, Levallois S, Hautefort C, Michel V, Larrous F, Verillaud B, Aparicio C, Wagner S, Gheusi G, Kergoat L, Kornobis E, Donati F, Cokelaer T, Hervochon R, Madec Y, Roze E, Salmon D, Bourhy H, Lecuit M, Lledo P-M. COVID-19-related anosmia is associated with viral persistence and inflammation in human olfactory epithelium and brain infection in hamsters. Science Translational Medicine. 2021;13 (596):eabf8396.<br>2. de Melo GD, Lazarini F, Larrous F, Feige L, Kornobis E, Levallois S, Marchio A, Kergoat L, Hardy D, Cokelaer T, Pineau P, Lecuit M, Lledo P-M, Changeux J-P, Bourhy H. Attenuation of clinical and immunological outcomes during SARS-CoV-2 infection by ivermectin. EMBO Mol Med. 2021;13:e14122.
Data exclusions	No data were excluded.
Replication	In vivo experiments: 8 animals in each group (2 independent replicates with 4 animals each; all attempts at replication were successful)<br>in vitro experiments: 3 independent replicates (all attempts at replication were successful)
Randomization	In vivo: there was no randomization due to pre-defined housing conditions (separeted isolators between infected and non-infeted animals).<br>In vitro: cell culture plates and chambers were randomly assigned at the start of each experiment.
Blinding	Blinding was not possible during in vivo experiments because each experimental group of animals was housed in dedicated isolators. However, ex vivo and in vitro analysis were blinded (an alphanumeric code was assigned to the samples).

Reporting for specific materials, systems and methods

We require information from authors about some types of materials, experimental systems and methods used in many studies. Here, indicate whether each material, system or method listed is relevant to your study. If you are not sure if a list item applies to your research, read the appropriate section before selecting a response.

Materials & experimental systems

n/a	Involved in the study
☐	☒ Antibodies
☐	☒ Eukaryotic cell lines
☒	☐ Palaeontology and archaeology
☐	☒ Animals and other organisms
☒	☐ Clinical data
☒	☐ Dual use research of concern

Methods

n/a	Involved in the study
☒	☐ ChIP-seq
☒	☐ Flow cytometry
☒	☐ MRI-based neuroimaging

Antibodies

Antibodies used

Rabbit anti-SARS CoV2 nucleocapsid antibody (GTX135357, GeneTex) 1:1000
Goat anti-CD31 (AF3628, R&D Systems) 1:300
Rat anti-Podocalyxin (MAB1556, R&D Systems) 1:1000
Donkey anti-Rabbit Alexa 555 (A-31572, Thermo Fisher Scientific) 1:500
Donkey anti-Goat Alexa 647 (A-21447, Thermo Fisher Scientific) 1:500
Chicken anti-Rat Alexa 647 (A-21472, Thermo Fisher Scientific) 1:500
SARS Nucleocapsid Protein antibody (NB100-56576, Novus Biologicals) 1:500
Goat anti-rabbit Ig secondary antibody (E0432, Dako, Agilent) 1:600
Mouse anti-β-Tubulin III (neuronal) antibody (T8578, Sigma-Aldrich) 1:1000
Goat anti-mouse AlexaFluor 647 (A21235, Invitrogen) 1:1000
Goat anti-rabbit AlexaFluor 546 (A11035, Invitrogen) 1:1000

Validation

All antibodies are commercially available and validated by the manufacturers or by refereed articles cited on each supplier's website.

Eukaryotic cell lines

Policy information about cell lines and Sex and Gender in Research

Cell line source(s)

Vero E6 (ATCC #CRL-1586)
A549 cells expressing ACE2 and TMPRSS2 (kindly provided by Pr. Olivier Schwartz, Institut Pasteur)
Human neural stem cells (hNSC, ENStem-A, SCC003, EMD-Millipore) for neuron differentiation

Authentication

Cell lines were not authenticated.
Neurons were identified by immunofluorescence with b3-tubulin marker after 14 days of differantiation from hNSCs.

Mycoplasma contamination

Cell lines were not tested

Commonly misidentified lines (See ICLAC register)

No commonly misidentified cell lines were used in the study

Animals and other research organisms

Policy information about studies involving animals; ARRIVE guidelines recommended for reporting animal research, and Sex and Gender in Research

Laboratory animals

Golden Syrian hamsters (Mesocricetus auratus; RjHan:AURA) of 5-6 weeks of age (average weight 60-80 grams)

Wild animals

No wild animals were used in the study.

Reporting on sex

Males were used as they are more sensitive to the disease and to olfaction impairments (https://www.embopress.org/doi/full/10.15252/emmm.202114122). Exceptionally, three female hamsters were added (in addition to three males) in the light-sheet imaging experiments to visualize the infection in the olfactory pathway.

Field-collected samples

No field collected samples were used in the study.

Ethics oversight

All animal experiments were performed according to the French legislation and in compliance with the European Communities Council Directives (2010/63/UE, French Law 2013–118, February 6, 2013) and according to the regulations of Institut Pasteur Animal Care Committees. The Animal Experimentation Ethics Committee (CETEA 89) of the Institut Pasteur approved this study (200023; APAFIS#25326-2020050617114340 v2) before experiments were initiated. Hamsters were housed by groups of 4 animals in isolators and manipulated in class III safety cabinets in the Pasteur Institute animal facilities accredited by the French Ministry of Agriculture for performing experiments on live rodents. All animals were handled in strict accordance with good animal practice.

Note that full information on the approval of the study protocol must also be provided in the manuscript.
